# Density assessment and reporting for *Phlebotomus perniciosus* and other sand fly species in periurban residential estates in Spain

**DOI:** 10.1007/s00436-021-07270-0

**Published:** 2021-08-18

**Authors:** C. Muñoz, J. Risueño, P. Pérez-Cutillas, L. J. Bernal, J. M. Ortiz, R. Ruiz de Ybáñez, P. F. Sánchez-López, C. Martínez-Carrasco, L. Del Río, P. De la Rúa, J. D. García-Martínez, M. Gonzálvez, L. Murcia, F. Collantes, E. Goyena, T. Spitzova, S. Elshanat, E. Berriatua

**Affiliations:** 1 Departamento de Sanidad Animal, Facultad de Veterinaria, Regional Campus of International Excellence “Campus Mare Nostrum”, Universidad de Murcia, Murcia, Spain; 2 Departamento de Geografía, Universidad de Murcia, Murcia, Spain; 3 Departamento de Medicina Y Cirugía Animal, Facultad de Veterinaria, Regional Campus of International Excellence “Campus Mare Nostrum”, Universidad de Murcia, Murcia, Spain; 4 Servicio de Sanidad Ambiental, Dirección General de Salud Pública Y Adicciones, Consejería de Salud de La Región de Murcia, Murcia, Spain; 5 Área de Biología Animal, Departamento de Zoología Y Antropología Física, Facultad de Veterinaria, Regional Campus of International Excellence “Campus Mare Nostrum”, Universidad de Murcia, Murcia, Spain; 6 Departamento de Genética Y Microbiología, Facultad de Medicina, Regional Campus of International Excellence “Campus Mare Nostrum”, Universidad de Murcia, Murcia, Spain; 7 Departamento de Zoología Y Antropología Física, Facultad de Biología, Regional Campus of International Excellence “Campus Mare Nostrum”, Universidad de Murcia, Murcia, Spain; 8 Department of Parasitology, Faculty of Science, Charles University, Vinicna 7, 128 44, Prague 2, Czech Republic; 9 Department of Parasitology, Faculty of Veterinary Medicine, Alexandria University, Alexandria, Egypt

**Keywords:** *Phlebotomus*, Distribution, Density, Environment, Residential, *Leishmania*

## Abstract

Green periurban residential areas in Mediterranean countries have flourished in the last decades and become foci for leishmaniasis. To remedy the absence of information on vector ecology in these environments, we examined phlebotomine sand fly distribution in 29 sites in Murcia City over a 3-year period, including the plots of 20 detached houses and nine non-urbanized sites nearby. We collected 5,066 specimens from five species using “sticky” interception and light attraction traps. The relative frequency of the main *Leishmania infantum* vector *Phlebotomus perniciosus* in these traps was 32% and 63%, respectively. Sand fly density was widely variable spatially and temporally and greatest in non-urbanized sites, particularly in caves and abandoned buildings close to domestic animal holdings. *Phlebotomus perniciosus* density in house plots was positively correlated with those in non-urbanized sites, greatest in larger properties with extensive vegetation and non-permanently lived, but not associated to dog presence or a history of canine leishmaniasis. Within house plots, sand fly density was highest in traps closest to walls. Furthermore, the study provides a guideline for insect density assessment and reporting and is envisioned as a building block towards the development of a pan-European database for robust investigation of environmental determinants of sand fly distribution.

**Supplementary Information:**

The online version contains supplementary material available at 10.1007/s00436-021-07270-0.

## Introduction

Phlebotomine sand flies (Diptera: Psychodidae) are endemic in tropical and subtropical latitudes as well as the Mediterranean subregion, where they are vectors of life-threatening *Leishmania* spp. (Kinetoplastida: Trypanosomatidae) and arboviruses (*Phlebovirus*,* Vesiculovirus*, and *Orbivirus*) (Akhoundi et al. 2016). *Leishmania infantum* is the only endemic species in Spain, and it causes zoonotic visceral leishmaniasis, a major disease of dogs with a considerable public health impact (Herrador et al. 2015; Gálvez et al. 2020). Phleboviruses identified in Spain include Toscana, Granada, Naples, Sicily, Arbia, and Arrabida-like viruses, and the risk of infection is considered moderate for Toscana virus and low for the other viruses (García San Miguel et al. 2020). Among the twelve sand fly species described in Spain (Gil Collado et al. 1989; Martínez Ortega et al. 1992), *Phlebotomus perniciosus* and *Phlebotomus ariasi* are vectors of *L. infantum*, and the former is the predominant species in southeast Spain (Risueño et al. 2017). Sand flies are considered to have low specificity for Phleboviruses (Ayhan and Charrel 2017), and six viral isolates were detected in *P. perniciosus* in a human leishmaniasis outbreak in a residential area in the outskirts of Madrid (Arce et al. 2013; Remoli et al. 2016). This unprecedented outbreak highlights the potential risks of leishmaniasis associated with environmental changes in the natural environment of sand fly vectors. It was the result of housing developments in former agricultural land leading to a massive buildup of *P. perniciosus*, coinciding with a demographic explosion of leporids (*Lepus granatensis* and *Oryctolagus cuniculus*) on which they fed and behaved as an unusual reservoir of *L. infantum* (Molina et al. 2012; Arce et al. 2013). Human and canine leishmaniasis (CanL) are also emerging infections in modern residential estates built in the outskirts of cities, consisting of detached and semidetached family houses with a garden and dogs (Pérez-Cutillas et al. 2015; Goyena et al. 2016), which are the main domestic reservoir of *L. infantum* (WHO 2010). It is logical to assume that these places provide ideal environments for sand fly vectors including sites protected from desiccation and with abundant organic material for sand flies to breed and rest, as well as a close-by source of blood required by females for egg development (Alexander, 2000). However, the precise locations are not well characterized, and to the best of our knowledge, there are no studies describing vector density and its relationship with environmental variables from these periurban settings.

Field investigations aiming for a representative picture of sand fly density and diversity in a particular area are expensive and difficult to perform. Their distribution is seasonal and highly heterogeneous at fine geographical scales, requiring a large sampling effort (Rioux et al. 2013; Muñoz et al. 2018, 2019). Moreover, there is no universal methodological guideline for estimating and reporting sand fly density, i.e., sand fly numbers in relation to sampling effort. In this sense, entomologists make use of a wide variety of trapping methods and protocols; results are often difficult or impossible to compare across studies; and published data may not be sufficient for other researchers to perform wider scale quantitative analysis. The first objective of the present study is to provide an insight into the spatial distribution of *P. perniciosus* and other sand fly species in periurban residential properties located in the outskirts of Murcia City (southeast Spain). Our second objective includes a proposal about the type of data that should be reported in a scientific journal to allow meta-analytic investigations of the environmental factors that affect sand fly temporal and spatial distribution. Such investigations are essential to improve our understanding and capacity to prevent and control vector-borne infections (https://www.ecdc.europa.eu/en/about-us/partnerships-and-networks/disease-and-laboratory-networks/vector-net).

## Materials and methods

### Study area and design

**Fig. 1:**
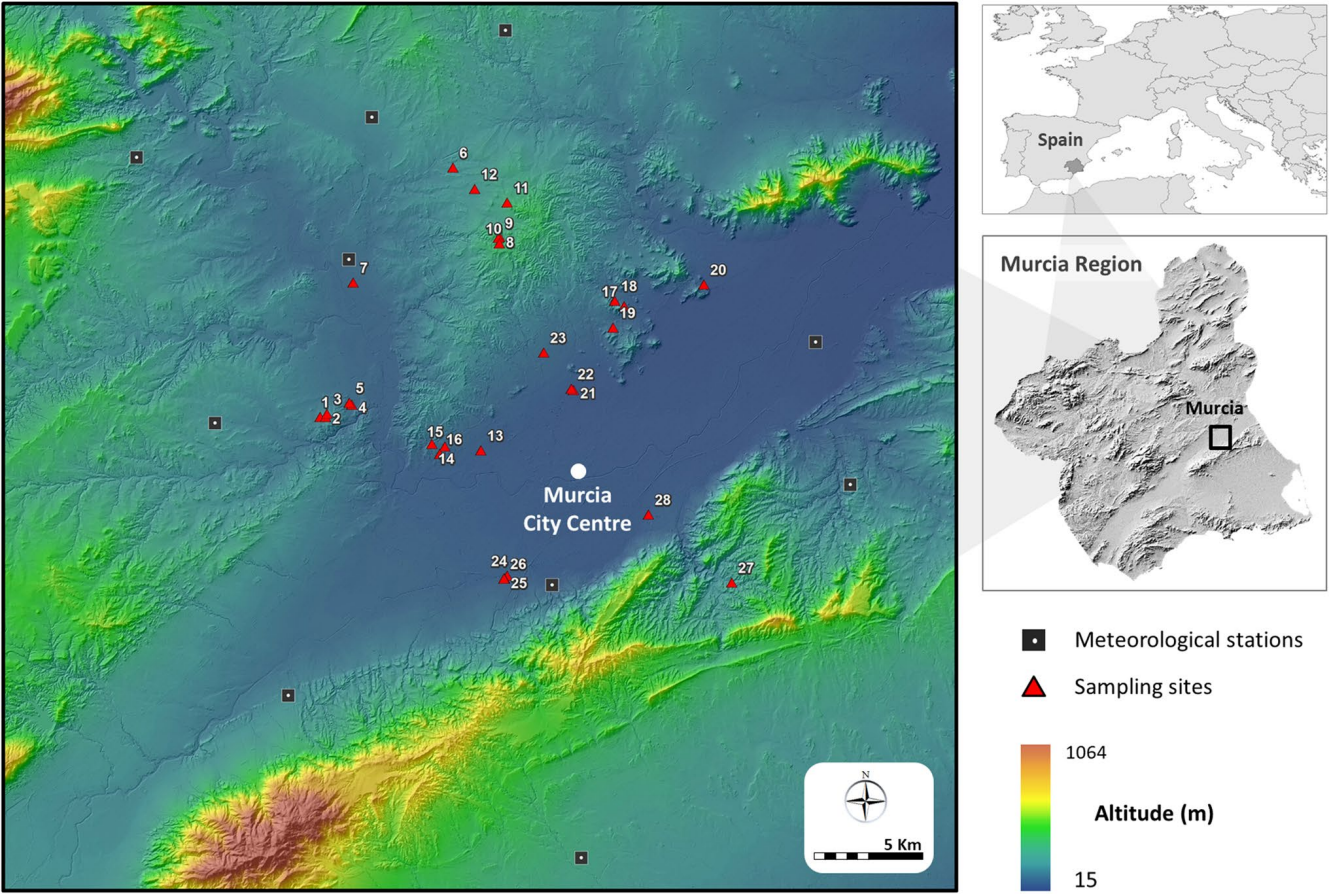
Location of phlebotomine sand fly sampling sites in periurban areas of Murcia City and meteorological stations from which climatic data was obtained

Murcia City has 453,000 inhabitants and is located in a region endemic for sand flies and sand fly-borne infections, both in dogs and humans (Martínez-García et al. 2007; Pérez-Cutillas et al. 2015; Goyena et al. 2016; Muñoz et al. 2019). Sand fly sampling was performed in 29 sites in 13 housing estates. They were located four to 14 km from the city center, except one site which was 26 km away. Sampling was performed in the summers of 2013, 2014, and 2015 during 25 weeks (see below). Sites included the outside plots (mainly gardens) of 20 detached houses and nine non-urbanized sites situated in the periphery of the housing estates (Fig. 1, Table 1). The latter were included to monitor the degree of sand fly threat to which the housing estates were potentially exposed. Due to limited resources, it was not possible to sample non-urbanized sites in every estate, and those selected were a representative example of the non-urbanized landscape in this part of Murcia. Houses were conveniently selected as they belonged to the research team families and other trustworthy people, in an attempt to ensure long-term adherence to the study. Eleven houses had one or more dogs, four had had CanL cases in the previous five years, and all participants knew of CanL cases in the neighborhood.

**Table 1:** Percentage of sticky and light traps with sand flies and sand fly density in 29 periurban sites in Murcia City in 2013–2015

Estate/site	Environment	Latitude/longitude (zone 30S)	Sticky traps							CDC light traps			
			No. of weeks	No. of traps	% positive traps	No. of sand flies	Trap area (m^2^)	Sampling effort^a^	Sand fly density^b^	No. of traps^c^	% positive traps	No. of sand flies	Sand fly density^2^
1/1	Non-urban^d^	653667.7/4208231.8	12	74	93	751	4.6	32.3	23.2	-	-	-	-
1/2	House plot	653914.4/4208225.3	20	285	40	335	17.8	124.5	2.7	4	100	25	6.3
1/3	House plot	653967.9/4208363.3	20	245	42	307	15.3	104.0	3.0	4	100	15	3.8
1/4	House plot	655073.0/4208773.0	20	239	26	208	14.9	103.8	2.0	4	100	49	12.3
1/5	House plot	654948.2/4208851.9	14	149	19	49	9.3	66.3	0.7	3	100	47	15.7
2/6	Non-urban	659529.9/4219233.3	12	142	42	483	8.9	67.3	7.2	4	100	12	3.0
3/7	House plot	655131.2/4214164.0	12	104	32	56	6.6	50.2	1.1	4	100	15	3.8
4/8	Non-urban	661587.4/4216176.4	15	162	7	15	10.1	69.1	0.2	-	-	-	-
4/9	House plot	661533.9/4216140.4	16	108	18	26	6.7	46.1	0.6	5	100	13	2.6
4/10	House plot	661581.3/4215885.7	12	84	12	10	5.2	35.9	0.3	4	100	14	3.5
4/11	Non-urban	661917.0/4217693.0	11	72	7	7	4.5	31.4	0.2	-	-	-	-
4/12	House plot	660487.9/4218302.0	11	58	21	21	3.6	25.4	0.8	3	100	11	3.7
5/13	House plot	660762.8/4206747.6	4	32	28	64	2.0	14.0	4.6	-	-	-	-
5/14	Non-urban	658596.3/4207038.2	11	96	38	78	6.0	41.1	1.9	-	-	-	-
5/15	House plot	659167.9/4206919.6	19	140	3	4	8.7	59.5	0.1	4	25	1	0.3
5/16	House plot	658946.7/4206592.0	8	53	19	21	3.3	22.0	1.0	-	-	-	-
6/17	Non-urban	667080.8/4213124.3	5	48	44	297	3.2	22.7	13.1	-	-	-	-
6/18	House plot	666660.6/4213356.0	5	50	42	115	3.7	26.2	4.4	2	100	59	29.5
9/19	House plot	666589.4/4212170.4	10	110	12	18	6.9	48.0	0.4	3	67	4	1.3
7/20	House plot	670594.1/4214073.1	12	155	43	146	9.7	68.3	2.1	4	100	115	28.8
8/21	Non-urban	664857.6/4209411.4	10	86	17	28	5.4	42.1	0.7	-	-	-	-
8/22	House plot	664752.1/4209476.1	17	217	62	835	13.5	101.0	8.3	4	100	16	4.0
10/23	House plot	663540.0/4211065.0	3	15	7	1	0.9	6.6	0.2	-	-	-	-
11/24	Non-urban	661913.0/4201210.0	12	75	20	22	4.7	32.8	0.7	-	-	-	-
11/25	House plot	661809.0/4201088.0	12	78	4	4	4.9	34.1	0.1	4	0	0	0.0
11/26	House plot	661777.0/4201090.0	12	74	0	0	4.6	32.3	0.0	4	50	2	0.5
12/27	Non-urban	671824.9/4200912.1	12	68	15	20	4.2	29.8	0.7	-	-	-	-
12/28	House plot	668160.0/4203927.0	18	234	58	656	14.6	100.9	6.5	4	100	75	18.8
13/29	House plot	687342.0/4218749.0	12	75	9	9	4.7	33.5	0.3	3	67	7	2.3
All				3328	31	4586	208.7	1471	3.1	67	84	480	7.2

^a^Sampling effort, the number of trapping days multiplied by the trap area (m^2^) for sticky traps and by the number of traps in the case of light traps

^b^Sand fly density, the number of sand flies collected divided by the sampling effort

^c^In this case, the number of traps equals the sampling effort

^d^Non-urbanized areas situated in the perimeter of the residential estates where house plots were located

Sites were georeferenced using a submetric GPS (Trimble geoXH), using a differential correction through RINEX files provided by GNSS services close to the study area. Environmental temperature and relative humidity and wind speed were obtained to analyze its relationship with sand fly density as described below. Other factors analyzed, considered potentially associated with sand fly density, were the number and age of human and animal occupants in the house, house and plot sizes (vegetated and paved areas), and the presence of a swimming pool. Trap location features were also recorded, including orientation, sun, wind exposure (as presumed by owners), ground type, roof cover, and distance to the closest wall, firewood and stone piles, plants, water tab, stationary water and irrigation point, and presence of a dog house. The most common plants situated in the proximity of the trap were ivy, a variety of lawn grasses, citrus fruit trees, cypresses, Mediterranean pine, and bougainvillea. Shrubs were the predominant vegetation in non-urbanized sites including large extensions of rosemary and thyme.

### Sampling protocol and trap types

Sand flies were sampled for 25 weeks in four periods between September 2013 and July 2015: first period, from the 3rd week of September to the 2nd week of October 2013; second period, from the 4th week of May to the 2nd week of July 2014; third period, from the 2nd week in September to the 2nd week in October 2014; and fourth period, from the 4th week in May to the 4th week in July 2015. The number of sampling weeks varied between sites (Table 1), mainly due to volunteers dropping out before the study ended for personal reasons.

Interception traps, made of half an A4 sheet of tracing paper measuring 210 mm × 148.5 mm (except on very few occasions when the entire A4 sheets were used) impregnated with castor oil (“sticky traps”), were used throughout the study. The number of traps varied between 6 and 14 traps per site, with the aim of covering all potential main sand fly microhabitats in selected sites. Traps were individually identified, placed always in the same spot close to the ground, exposing both sides of the sheets to sand flies, and they were kept untouched for an average of 7 days/week (range, 4–14 days/week) and 94% of traps between 6 and 8 days/week.

Battery-operated, miniature Centers for Disease Control and Prevention (CDC) light attraction traps (J. W. Hock Company, Gainesville, FL, USA) were also used fortnightly in 18 house plots (one trap per house plot) in June and July 2015, making a total of two to five nights (from 8 am to 8 pm) in each of those plots. The aim was to compare species diversity detected in both trap types. Traps were placed close to vegetation and a wall, at approximately 1.5 m from the ground to avoid dog and cat interference.

### Sand fly identification

Sticky traps were collected and stored individually between two A4 paper sheets each and kept at 4 °C until sand flies were removed from the traps using a fine brush dipped in 70% ethanol. Collection cups from light traps were kept at − 20 °C for at least 2 h to kill the insects. Specimens from sticky and light traps were then maintained in 70% ethanol until morphologically identified based on the external genitalia in males and the pharynx, cibarium, and spermatheca in females (Martínez-Ortega and Conesa-Gallego 1987; Gállego-Berenguer et al. 1992). Male and female specimen preparation consisted of the dissection of the head and two terminal abdominal segments, clarification in Marc Andre solution, and mounting on a glass slide using Hoyer’s medium. Slides were examined under the microscope at × 400 magnification.

### Altitude and climatic data collection

Site altitude was obtained from the high-resolution (1 m per pixel) digital elevation model of the LIDAR project from the “Plan Nacional de Ortofotografía Aérea (PNOA)” (https://pnoa.ign.es) and ranged from 23 to 287 m above sea level (a.s.l.). Climatic real-time data was collected from ten close-range meteorological stations (http://siam.imida.es) (Fig. 1). They included the mean temperature and relative humidity (RH %) and the mean and maximum wind speed. ArcGIS v.10 (ESRI, Redlands, USA) was used to produce continuous map layers of these variables with values from the nightly periods (20.00 h to 8.00 h) when adult sand flies are most active and with a spatial resolution of 5 m/pixel. Site 29, situated far from the other sites, was excluded from this analysis. Wind speed was estimated using the inverse weighted distance interpolation method (Keskin et al. 2015). Temperature and RH layers were developed employing a linear regression model using residual-corrected altitude as the independent, explanatory variable. The validity of the estimated temperature and RH % was assessed by comparing the 2015 data with similar measurements taken at the same time from thermohygrometers (Digital Logtag Haxo-8 T, Templyzer) placed in 18 sampling sites, using Pearson’s correlation coefficient.

### Data description and statistical analysis

Abundance referred to the absolute number of sand flies, species richness was the number of different species, and species diversity considered both the number of different species and their relative frequency. “Positive traps” were those with at least one sand fly. “Sand fly density” was defined as the number of sand flies collected divided by the sampling effort. The “sampling effort” was established as the number of trapping days multiplied by the trap area (m^2^) for sticky traps and by the number of traps in the case of light traps. The proportion of positive traps and median sand fly densities across environmental explanatory variables were compared using Yates-corrected chi-squared test (or when necessary Fisher exact test) and Kruskal–Wallis test, respectively. Spearman’s rank coefficient test for non-normally distributed data was employed to evaluate the correlation between sand fly density in sticky and light traps and between house plots and non-urbanized sites situated within 500 m.

Separate multilevel negative binomial regression for overdispersed count data was developed to investigate site- and trap-level factors associated with *P. perniciosus* density in sticky traps, respectively. These models allowed an assessment of correlation in the insect’s density from repeated sampling of the same sites and trap locations over time (two-level hierarchical models with weeks as level 1 and sites or traps as level 2 random variables) (Snijders and Bosker 1999). To avoid potential bias resulting from analyzing data from sites sampled during different periods, modeling was performed in two data subsets. Subset “a” included the 5 weeks in September and October 2014 when all except three sites were continuously sampled, and subset “b” was all data from house plot sites 2, 3, 4, 22, and 28, which were the only ones sampled almost every week throughout the study and had a moderately high sand fly density. Subset “a” was used to investigate site-specific explanatory variables (climatic and others) that were associated with *P. perniciosus* density in the bivariate analysis and included site and weeks as random effects. With subset “b,” we investigated trap-level variables and which together with climatic variables were incorporated in the model as fixed effects and trap and weeks as random variables. In all cases, the decimal logarithm of the species density + 1 was the outcome variable, and a backward model building strategy was used including all fixed explanatory variables. Since some environmental variables were strongly correlated, for example, the highest altitude range included only small house plots and none of the largest house plots were permanently inhabited, Akaike’s information criteria was used to select models with different combinations of variables, choosing those with the lowest value (Kleinbaum et al. 1998). Models were estimated using the maximum likelihood method using the glmer.nb function in the lme4 package in R (https://cran.r-project.org/web/packages/lme4/lme4.pdf) (Bates et al. 2015). For all analysis, significance was confirmed when *α* = 5% (*p* < 0.05) for a two-tailed test.

## Results

### Sampling effort and sand fly abundance and density

A total of 3,498 sticky traps were placed for an average of 7 days/trap in 29 sites over 25 weeks between September 2013 and July 2015, and 3,328 (95%) traps were recovered, totaling 208.7 m^2^ of sticky trap surface. The resulting sampling effort was 1,471 m^2^ × days. Since 4,586 sand flies were collected in the sticky traps, with 31% of them being positive, the overall density was 3.1 sand flies/m^2^/day (smd) (Table 1). However, there were large differences between sites in all of these parameters. Sampling efforts ranged from 6.6 m^2^ × days in site 23 to 124.5 m^2^ × days in site 2, the percentage of positive traps went from 0% in site 26 to 93% in site 1; and sand fly density in the 28 positive sites ranged from 0.1 smd in sites 15 and 25 to 23.2 smd in site 1 (Table 1). Globally, the percentage of positive traps and sand fly density was significantly higher in non-urbanized areas compared to house plots, and there was a moderate correlation between sand fly density in non-urbanized areas and house plots situated within 500 m (rho = 0.45, *p* < 0.05).

CDC light traps placed in June and July 2015 in 18 sites represented 67 trap nights and collected 480 sand flies in 84% of the traps. Hence, sand fly density was 7.2 sand flies/trap/night (stn) overall and ranged between 0 and 29.5 stn in sites 25 and 18, respectively (Table 1). There was a positive correlation between sand fly density measured by sticky and light traps in all study sites (rho = 0.71, *p* < 0.05).

### Sand fly species frequency and abundance

The number (relative frequency) of sand flies identified to species level from sticky traps was 4,464 (97%), of which 65% were males and 35% were females. The relative frequencies (male/female ratio) of species included *Sergentomyia minuta*, 60 (46/54)%; *P. perniciosus*, 32 (92/8)%; *Phlebotomus papatasi*, 5 (90/10)%; *Phlebotomus sergenti*, 3 (94/6)%; and *P. ariasi*, 0.4 (88/12)% (Table 2). The remarkable difference in the sex ratio between *S. minuta* and other species was observed in most places (Table 2). The relative abundance of species varied between sites; for example, in sites where sand flies were most abundant, *S. minuta* dominated in sites 2, 22, and 28, whereas *P. perniciosus* was comparatively more abundant in sites 4 and 17 (Table 2). Similarly, *P. papatasi* was relatively more abundant in sites 1 and 18 and *P. sergenti* in 28, compared to other sites (Table 2).

**Table 2:** Absolute number of sand flies identified and relative frequency (male/female ratio) of species in sticky and CDC light traps in study sites in 29 periurban sites in Murcia’s metropolitan area in 2013–2015

Site	No. of sand flies		*P. ariasi*		*S. minuta*		*P. papatasi*		*P. perniciosus*		*P. sergenti*	
	Sticky	Light	Sticky	Light	Sticky	Light	Sticky	Light	Sticky	Light	Sticky	Light
1	745	0	0.8 (100/0)	-	51 (44/56)	-	14 (95/5)	-	32 (96/4)	-	2 (89/11)	-
2	323	24	0	0	78 (41/59)	8 (50/50)	3 (89/11)	25 (0/100)	19 (93/7)	67 (12/88)	0	0
3	297	14	0	0	56 (44/56)	43 (50/50)	3 (80/20)	14 (50/50)	41 (91/9)	36 (40/60)	0	7 (0/100)
4	205	48	0.5 (100/0)	2 (0/100)	34 (64/36)	21 (60/40)	0.5 (100/0)	2 (0/100)	65 (99/1)	75 (58/42)	0.5 (100/0)	0
5	43	46	0	0	26 (36/64)	11 (100/0)	0	4 (0/100)	67 (93/7)	85 (62/38)	7 (100/0)	0
6	480	12	0	0	58 (51/49)	58 (71/29)	1 (100/0)	0	41 (87/13)	42 (20/80)	0	0
7	55	15	0	0	69 (58/42)	20 (100/0)	13 (57/43)	27 (0/100)	18 (90/10)	53 (25/75)	0	0
8	14	0	0	-	86 (33/67)	-	0	-	14 (100/0)	-	0	-
9	25	13	0	0	72 (83/17)	15 (50/50)	0	8 (0/100)	28 (86/14)	77 (70/30)	0	0
10	10	13	0	0	80 (38/63)	8 (100/0)	0	0	20 (100/0)	92 (42/58)	0	0
11	7	0	0	-	57 (50/50)	-	0	-	43 (100/0)	-	0	-
12	20	11	0	0	75 (40/60)	9 (100/0)	0	0	25 (60/40)	91 (30/70)	0	0
13	61	0	0	-	80 (47/53)	-	0	-	20 (92/8)	-	0	-
14	78	0	0	-	81 (56/44)	-	4 (100/0)	-	14 (82/18)	-	1 (0/100)	-
15	4	1	0	0	75 (67/33)	100 (100/0)	0	0	25 (100/0)	0	0	0
16	19	0	0	-	11 (50/50)	-	0	-	84 (100/0)	-	5 (100/0)	-
17	296	0	0.7 (100/0)	-	24 (26/74)	-	0.4 (100/0)	-	75 (95/5)	-	0	-
18	115	59	2 (50/50)	5 (33/66)	0	10 (50/50)	24 (86/14)	12 (29/71)	70 (90/10)	71 (67/33)	3 (100/0)	2 (100/0)
19	18	4	0	0	67 (42/58)	25 (0/100)	6 (100/0)	0	28 (80/20)	75 (33/67)	0	0
20	145	115	4 (83/17)	17 (55/45)	39 (54/46)	25 (62/38)	10 (73/27)	6 (57/43)	45 (75/25)	51 (81/19)	2 (100/0)	0
21	28	0	0	-	89 (76/24)	-	4 (0/100)	-	7 (50/50)	-	0	-
22	794	16	0	0	88 (35/65)	38 (50/50)	1 (80/20)	6 (0/100)	11 (93/7)	56 (67/33)	0.3 (50/50)	0
23	1	0	0	-	100 (0/100)	-	0	-	0	-	0	-
24	22	0	0	-	68 (87/13)	-	9 (100/0)	-	23 (100/0)	-	0	-
25	4	0	0	0	100 (100/0)	0	0	0	0	0	0	0
26	0	2	0	0	0 (0/9)	0	0	0	0	100 (50/50)	0	0
27	20	0	0	-	45 (100/0)	-	0	-	55 (100/0)	-	0	-
28	628	74	0	0	67 (56/44)	42 (58/42)	2 (100/0)	12 (22/78)	17 (94/6)	46 (53/47)	14 (97/3)	0
29	7	7	0	0	43 (33/67)	0	29 (50/50)	0	29 (50/50)	100 (14/86)	0	0
All	4464	474	0.4 (88/12)	5 (50/50)	60 (46/54)	24 (62/38)	5 (90/10)	8 (23/78)	32 (92/8)	63 (57/43)	3 (94/6)	0.4(50/50)

CDC light trap specimens identified at species level included 474 sand flies (99%) with 55% males and 45% females. The same five species from sticky traps were detected in the light trap, but their relative frequency and sex ratio was very different to the former. Species percentages (male/female) were *P. perniciosus*, 63 (57/43)%; *S. minuta*, 24 (62/38)%; *P. papatasi*, 8 (23/78)%; *P. ariasi*, 5 (50/50)%; and *P. sergenti*, 0.4 (50/50)%. The relative abundance of light trap species also differed according to site. In the five sites where sand flies were most abundant (sites 4, 5, 18, 20, and 28), species proportions ranged from 46–85% for *P. perniciosus*, 10–42% for *S. minuta*, 2–12% for *P. papatasi*, 0–17% for *P. ariasi*, and 0–2% for *P. sergenti* (Table 2).

### Sand fly temporal dynamics and relationship with climatic variables

The proportion of positive sticky traps and overall sand fly and *S. minuta* density in these traps peaked in September 2013 and in May and July 2014 (Table 3). Notably, sampling was not possible during these 3 months in sites 1, 6, and 17 which were among those with the highest sand fly density in the study (Table 1). In contrast, *P. perniciosus* density peaked in September 2013 and 2014. *Phlebotomus papatasi* and *P. sergenti* were found in low numbers most months although the majority of *P. sergenti* was captured in June 2014. In contrast, *P. ariasi* was detected only in September and October 2014 and May and July 2015 (Table 3). Sand fly density in CDC traps placed in June and July 2015 was also variable in time; total sand fly and *P. perniciosus* density peaked in the third week of July, while *S. minuta* density was highest in the first week of July (data not shown).

**Table 3:** Percentage of sticky traps with sand flies (positive traps) and sand fly density (No. sand flies/m^2^/day) according to month and year in 29 periurban sites in Murcia City

Year-month	No. of traps	% positive traps	Sampling effort^a^	No. of sand flies	Sand fly density^b^					
					All	*S. minuta*	*P. perniciosus*	*P. papatasi*	*P. sergenti*	*P. ariasi*
2013										
September	186	42	81	386	4.8	3.2	1.5	0.09	0.01	0.00
October	125	26	52	79	1.5	1.0	0.4	0.06	0.02	0.00
2014										
May	94	47	37	180	4.8	3.6	1.1	0.05	0.05	0.00
June	255	34	111	441	4.0	2.9	0.6	0.04	0.48	0.00
July	110	36	46	227	4.9	4.2	0.4	0.02	0.28	0.00
September	654	27	290	952	3.3	1.4	1.6	0.18	0.03	0.02
October	432	25	190	432	2.3	0.9	1.2	0.14	0.03	0.04
2015										
May	364	31	166	400	2.4	1.3	0.8	0.17	0.04	0.01
June	741	32	331	956	2.9	1.9	0.7	0.18	0.08	0.00
July	367	32	165	411	2.5	1.8	0.5	0.15	0.02	0.01

^a^Sampling effort, the number of trapping days multiplied by the trap area (m^2^)

^b^Sand fly density, the number of sand flies collected divided by the sampling effort

**Fig. 2:**
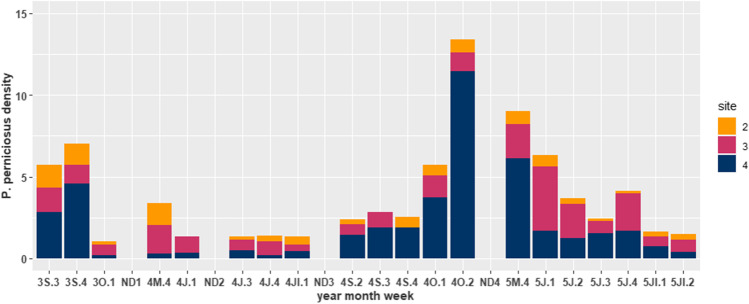
Temporal dynamics in *P. perniciosus* density (sand flies/m^2^/day) in sticky traps from week 3 in September 2013 (3S.3) to week 2 in July 2015 (5Jl.2) in the plot of three detached homes (sites 2, 3, and 4) in residential estate number 1, in the outskirts of Murcia City (southeast Spain). ND1-4 denote periods between two sampling weeks when no data was collected

The contrasting temporal dynamics between species, years, and sites in sticky traps are reflected in the weekly variation of *P. perniciosus* and *S. minuta* densities in sites 2, 3, and 4, situated in the same residential estate and sampled in the same weeks. In contrast to the overall pattern, *P. perniciosus* density peaked in the fourth week of October 2014 and May 2015 and was most abundant in site 4 (Fig. 2). *Sergentomyia minuta* predominated in sites 2 and 3 until the fourth week of July although it was similarly abundant in site 4 later, peaking in September 2014.

Regarding climatic variables, the nightly mean (range) RH (%), temperature, and wind speed and the maximum (range) wind speed in all sites during the study period were 70 (45–95)%, 20 (14–25)°C, 0.7 (0.2–1.4)m/s, and 2.0 (0.9–3.1)m/s, respectively. The overall sand fly density in sticky traps was negatively associated with the mean RH (%) and positively with the mean temperature and mean and maximum wind speed (*p* < 0.05). However, differences in the mean and maximum wind speed between high- and low-density sites were numerically very small: 0.11 m/s and 0.17 m/s, respectively.

### Sand fly density in sticky traps and site and trap location environmental features

Ten of the thirteen trap locations with a median sand fly density > 10 smd were in non-urbanized sites. They included three underground caves (29–44 smd); the ruins of an old pig farm (4 traps, 11–36 smd); an abandoned small, brick dog house (26 smd); an old pile of firewood (14 smd); and a 30-cm-wide rock crevice (11 smd). The pig farm and one of the caves were part of site 1 and were 200–300 m away from two sheep farms and house plot sites 2 and 3. The other two caves and the crevice were 50–100 m away from house plot site 18 and other premises that had backyard chickens and sporting pigeons.

Bivariate analysis indicated that *P. perniciosus* densities in house plots were significantly associated with larger properties situated in the middle altitude range, with extensive vegetated and non-vegetated earth areas and not permanently inhabited but used mostly during weekends and summer periods and without a swimming pool (Table 4). Moreover, it was not associated with the permanent presence of dogs or to having a history of CanL. Similarly, *P. perniciosus* density within house plots was greatest in places situated at some distance from people’s transit, protected from rains and near walls (Table 4).

**Table 4:** Percentage (95% CI) of sticky traps with *P. perniciosus* (positive traps) and median (range) density (No. specimens/m^2^/day) in positive traps according to house plot and trap location variables. A study of sand fly abundance in 29 periurban sites in Murcia City in southeast Spain

Variable	No. of traps	*Phlebotomus perniciosus*	
		% positive traps (95% CI)	Median (range) sand fly density^a^
**(A) House plot**			
Altitude			
23–90	1934	14 (12–16)	2 (1–71)
112–180	968	23 (21–26)*	2 (1–96)
248–287	426	3 (1–4)	2 (2–5)
Vegetated/soil area (m^2^)			
40–100	780	8 (6–10)	2 (2–16)
140–320	713	14 (11–17)	2 (1–50)
450–999	519	20 (16–23)*	2 (1–23)
1824–9096	493	19 (16–23)	2 (2–23)
Permanently lived			
No	710	20 (17–23)*	2 (1–32)
Yes	1795	12 (10–13)	2 (1–50)
Swimming pool			
No	775	18 (16–21)*	2 (1–32)
Yes	1730	12 (11–14)	2 (2–50)
**(B) Trap location**			
Transit area			
No	1132	16 (14–18)*	2 (1–50)
Yes	1192	12 (10–20)	2 (1–32)
Wind exposure^b^			
No	545	16 (13–19)	2 (1–16)
Low	1100	15 (13–17)	2 (1–50)
Moderate-strong	834	13 (10–15)	2 (1–23)
Undercover			
No	1724	12 (11–14)	2 (1–50)
Yes	755	20 (17–23)*	2 (1–16)
Distance to wall (m)			
0.0–0.1	833	22 (19–25)*	2 (1–50)*
0.2–0.5	659	12 (9–14)	2 (2–18)
0.8–2.0	499	9 (7–12)	2 (1–14)
2.5–10	432	11 (8–14)	2 (2–23)
> 10	56	4 (0–8)	2 (2–2)
Distance to soil/plants (m)			
0–0.5	1244	15 (13–17)	2 (1–50)*
0.75–2	517	13 (10–16)	2 (2–16)
3–10	527	17 (14–21)	2 (1–23)
15–40	191	10 (6–15)	2 (2–7)
Distance to dog sleeping area (m)			
0–5	346	19 (15–23)	2 (2–21)
6–20	607	17 (14–20)	3 (2–50)*
> 25	540	14 (11–17)	2 (2–23)

^*^p < 0.05. Asterisk placed in the highest median or maximum

^a^Sand fly density, the number of sand flies collected divided by the sampling effort, which is No. of trapping days multiplied by the trap area (m^2^)

^b^Wind exposure as presumed by owners

In the site-specific multilevel negative binomial model type “a,” none of the fixed explanatory variable were significantly associated with *P. perniciosus* density, and there was a large remaining, unexplained variability between sites (house plot variance, 46.79) and very little between weeks (0.03) (not tabulated). Instead, trap-level type “b” model indicated that *P. perniciosus* density decreased with trap distance to the wall and differed between sites, and there was some remaining unaccounted for variation between traps (trap variance, 1.17) (Table 5).

**Table 5:** Estimates from multilevel negative binomial regression models investigating trap-level factors associated with *P. perniciosus* density (log_10_-transformed) in sticky traps. Five sites sampled for 21 weeks in 2013–2015

Variable	Levels	Estimate	Se^a^	*p* value
*Fixed effects*				
Intercept		− 1.19	0.38	0.0017
Distance to a wall (m)	0	0.00		
	0.1–0.3	− 0.34	0.49	0.4936
	0.5–1	− 1.06	0.49	0.0289
	1.5–5	− 1.30	0.52	0.0125
	6–20	− 2.44	0.77	0.0015
Site	2	0.00		
	3	0.91	0.49	0.0618
	4	0.92	0.61	0.1342
	22	1.78	0.55	0.0012
	28	0.93	0.54	0.0826
*Random effects variance*				
Trap		1.17		
Week		0.001		

^a^Standard error

## Discussion

The present study assessed for the first time sand fly small-scale distribution and species diversity in modern periurban residential estates in *L. infantum* endemic southern Europe. Sand flies, mostly *S. minuta* and *P. perniciosus*, were widespread in the area of study, but their density varied greatly between and within sites. Moreover, the temporal dynamics differed between species, years, and sites situated close to each other. Also, as previously shown, the estimated species diversity and sex ratios of the five sand fly species here identified strongly depended on the trap type used (Martínez-Ortega 1985a, Martínez-Ortega et al. 1991; Alexander 2000; Alten et al. 2015; Muñoz et al. 2018).

The predominance of *S. minuta* and *P. perniciosus* is in agreement with other studies in Spain employing sticky traps (Gálvez et al. 2010; Ballart et al. 2014), and *P. ariasi*, *P. papatasi*, and *P. sergenti* are the other most frequently reported in studies in southeast Spain (Martínez-Ortega 1985b; Martínez-Ortega et al. 1991; Muñoz et al. 2018, 2019). Other species previously reported in southeast Spain in very small numbers and not detected in the present include *P. longicuspis*, *P. chabaudi*, *P. alexandri*, and *P. langeroni* (Martínez-Ortega 1985b; Martínez Ortega et al. 1992; Risueño et al. 2017; Díaz Sáez et al. 2018). *Phlebotomus longicuspis* is morphologically very similar to *P. perniciosus*, and specimens from Spain were proposed to be the same species (Collantes and Martínez Ortega 1997; Martín-Sánchez et al. 2000). *Phlebotomus langeroni* is typically found associated to rabbit burrows (Martínez Ortega et al. 1992; Díaz Sáez et al. 2018). *Phlebotomus chabaudi* is mostly found in Northern Africa (Lehrter et al. 2017). In contrast, *P. alexandri* and other closely related sister species have a wider distribution ranging from Morocco in the west to China in the East and Ethiopia in the south (Depaquit et al. 2000).

Comparatively, few premise-level environmental factors were associated with *P. perniciosus* density in the bivariate analysis and none in the multilevel modeling which highlighted large, unexplained variation between sites. This may reflect low statistical power, probably because relatively few sites from a fairly small and environmentally similar area were examined for unequal periods of time in some cases. Clearly, the results from the present study indicate that a larger sample size would be required to identify other features of periurban residencies that influence sand fly densities. They also highlight the need for a combined effort to survey multiple similar residential areas across Mediterranean countries using standard sampling and reporting methodology and the need for meta-analysis.

The precise locations where sand flies breed have not been fully characterized, and eggs, larvae, and pupae are very difficult to find in soil samples. As a result, most entomological surveys focus only on adult stages. In the natural environment, they are typically found resting in large numbers in places protected from desiccation such as caves, uninhabited buildings, rock crevices and undisturbed rock, and log piles close to groups of domestic animals (Alexander 2000). These were the precise habitats in the non-urbanized sites in the present study where sand flies were most abundant. Sand fly density in non-urbanized sites was positively correlated to that in close-by residencies, suggesting that the former could be a source of insects for the latter. This phenomenon is described in Israel, where residential areas are continuously exposed to *P. papatasi* from surrounding agricultural land (Orshan et al. 2016). Alcover et al. (2014) in Majorca similarly found greater *P. perniciosus* density at the edge compared to within human settlements. Further studies including a larger number of sites are necessary for a better understanding on how non-urbanized areas contribute to the vector population in nearby residential estates in Mediterranean countries.

Most likely, periurban residential estates also provided suitable breeding habitats for sand flies, and density was highly variable between traps in the same site. Sand fly populations are typically spatially overdispersed on a large and small geographical scale (Rioux et al. 2013; Alten et al. 2016; Muñoz et al. 2019). Like in previous studies, the density of *P. perniciosus* was negatively associated to trap distance to a wall (Risueño et al. 2017; Muñoz et al. 2018). Walls have several advantages for sand flies (Alexander 2000). They protect them from strong wind, and they often have vegetation growing at the base. Their surfaces allow them to rest and move vertically in typical short hopping steps, and holes and cracks provide suitable breeding places. However, other traps in the present study that were not situated close to walls also had high sand fly counts, but the multivariable analysis revealed no association between density and variables potentially affecting the insect’s life cycle such as being undercover or close to vegetation, surface water, or the dog’s sleeping place. Sticky traps are interception traps collecting a random and comprehensive selection of sand flies in the immediacy of the trap and are ideal for ecological studies investigating species diversity (Alexander 2000; Alten et al. 2015). Light trap captures are biased towards host-seeking phototrophic species present within a few meters (< 10 m) from the trap, including *P. perniciosus* and *P. ariasi L. infantum* vectors (Alexander 2000). However, neither sticky nor light traps inform on whether the site is suitable for breeding or not, and this constitutes an important limitation in sand fly and leishmaniasis control (Alexander 2000; Alten et al. 2015).

Climate determines the annual activity of sand flies, influencing the length of diapause during cold months, the number of life cycles, and the resulting adult density peaks between spring and autumn (Alten et al. 2016). *Phlebotomus perniciosus* seasonality in Murcia was found to be bimodal with maximum densities in July and September when using sticky traps (Martínez-Ortega 1986; Muñoz et al. 2018) and unimodal with a single July peak when sampling with CDC light traps (Muñoz et al. 2018). Here, the overall sand fly density differed between years, and three peaks were detected, one in September 2013, one in May 2014, and one in July 2014, and there were substantial differences between sites and species. All this reflects the complexity of the system regulating sand fly demographics at a small geographical scale, and that accurate estimation of species seasonality in a particular area requires continuous longitudinal sampling of a large number of sites over several years (Alten et al. 2016). In ideal laboratory conditions at 25–26 °C, *P. perniciosus* specimens from Murcia may take 41 to 47 days to complete a life cycle (Volf and Volfova 2011). Feeding preferences and attraction to light vary between species and sexes (Alexander 2000; Alten et al. 2015); while female *P. perniciosus* are highly phototropic and tend to concentrate closer to their blood source than males (Muñoz et al. 2018), *S. minuta* is less attracted to light, and in the rural environment, both male and females may be similarly abundant relatively far away from groups of hot-blooded animal groups, with females probably feeding on lizards (Muñoz et al. 2018). Such inherent biological diversity would also explain the remarkable sex- and trap-specific spatial and temporal distributional differences observed here and elsewhere (Martínez-Ortega 1985a; Martínez-Ortega et al. 1991; Muñoz et al. 2018). We can further conclude that a very large number of sticky traps are required to attain a representative picture of sand fly distribution in a particular site and gain statistical power to detect associations with environmental variables.

The small number of premises precluded a robust investigation of the relationship between leishmaniasis incidence and sand fly density, and it was not an objective of the present study. This issue is a matter of debate. Outbreaks are typically associated with large densities of infected vectors (Arce et al. 2013; Jiménez et al. 2013), but vectors may also be very abundant and infection prevalence low, in areas with a high density of non-*Leishmania* competent hosts (Muñoz et al. 2019). The need for a more in depth understanding of this highly relevant question is at the core of the VectorNet initiative (https://www.ecdc.europa.eu/en/about-us/partnerships-and-networks/disease-and-laboratory-networks/vector-net). The aim of this network of entomologists is to gather data on vectors related to both animal and human health, to generate maps and investigate environmental determinants of vector distributions (https://www.ecdc.europa.eu/sites/default/files/documents/vector-abundance-and-seasonality.pdf). Ideally, reports should convey quantitative sand fly density information at the insect species and sex level for each of the places sampled. Essential trap-related information includes the type, number, dimensions (for sticky traps), operational time, and precise geographical location. The number of consecutive days that the same traps are in operation is also an important parameter to consider (Gálvez et al. 2010). Sand fly population depletion and loss of viscosity in sticky traps over time may lead to an underestimation of sand fly density. In this study, relatively few traps per site were used and were placed in wide, open spaces, so it is very unlikely that sand flies were depleted from sites. Loss of trap adherence is particularly important in high humidity places (Alexander 2000), which is not the case of Murcia, and care was taken in the present study to impregnate the sheets thoroughly before using them. These issues require further investigation. Other useful data to report is on variables associated to the trap microenvironment including if the trap is protected from rain and wind and trap distance to the ground, to walls, and to resident animal groups (farms, kennels and catteries). These data should be incorporated into multivariable models to adjust sand fly species density estimations in a particular ecotope. In a recent review of published scientific studies reporting sand fly distribution data in Europe and neighboring countries, less than half of the articles provided the data needed to calculate the sampling effort and sand fly density (as here proposed), and this was particularly a problem when sticky traps were used (Muñoz C. and Berriatua E., personal communication). Other limitations with those studies included not providing precise geographical locations and scarce details on trap position relative to potential micro-environmental risk factors. We have been careful to provide all the information needed to ensure these data can be used in continental scale analyses. It is hoped that this paper will encourage other authors to provide such details when reporting the results of entomological surveys.

In summary, here we show that periurban residential estates provide the right conditions for sand fly vectors to thrive. We also proved that sand fly distribution is highly spatially temporarily heterogeneous at a very small geographical scale. Detailed understanding of factors governing sand fly density requires further studies with a similar reporting approach, which will enable a meta-analytic methodology to be implemented.

## Supplementary Information

Below is the link to the electronic supplementary material.

Supplementary file1 (PDF 1593 KB)

## Data Availability

The data that support the findings of this study are available from the corresponding author upon reasonable request.
