# Supplementary material for: Density assessment and reporting for Phlebotomus perniciosus and other sand fly species in periurban residential estates in Spain
Source: Parasitol Res. 2021 Aug 18;120(9):3091–103. doi: 10.1007/s00436-021-07270-0 (PMC8397643; doi:10.1007/s00436-021-07270-0)
Supplement: Supplementary file 1 — Supplementary file1 (PDF 1593 KB) [file 436_2021_7270_MOESM1_ESM.pdf]

Supplementary material

Photographs of places where sticky and light traps were placed. A study of sand fly abundance in 29 periurban sites in Murcia City in southeast Spain.

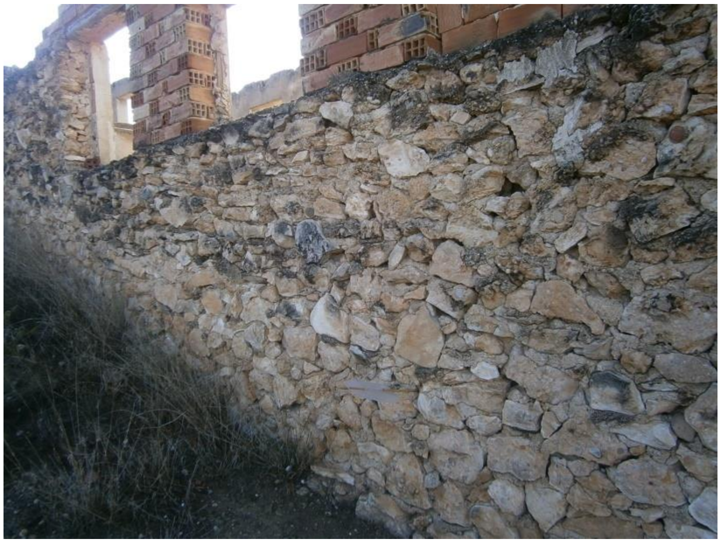

Photo 01: Stone wall in an abandoned pig farm (site 1).

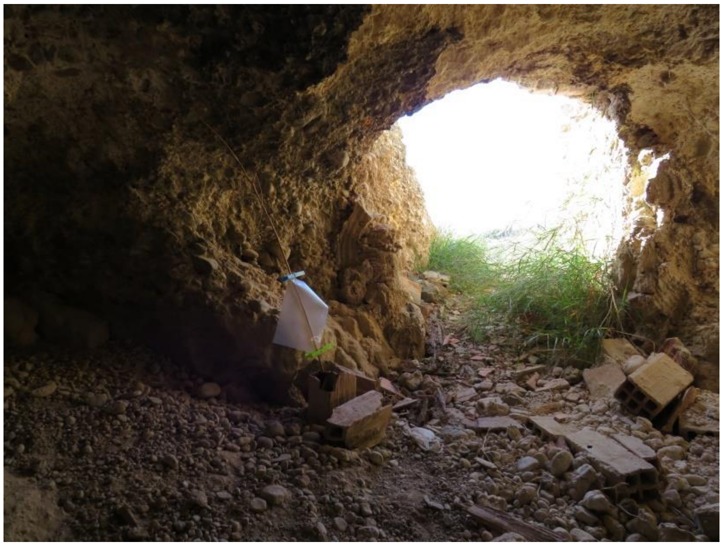

Photo 02: Open cave (site 1).

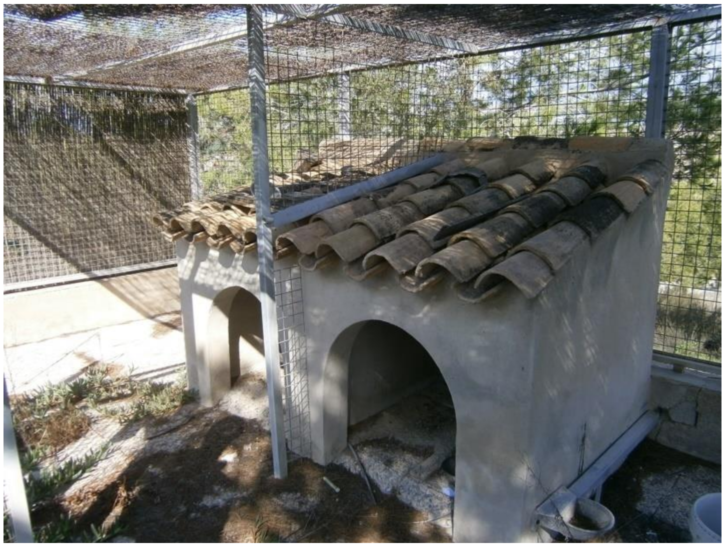

Photo 03: Abandoned small, brick dog house (site 6).

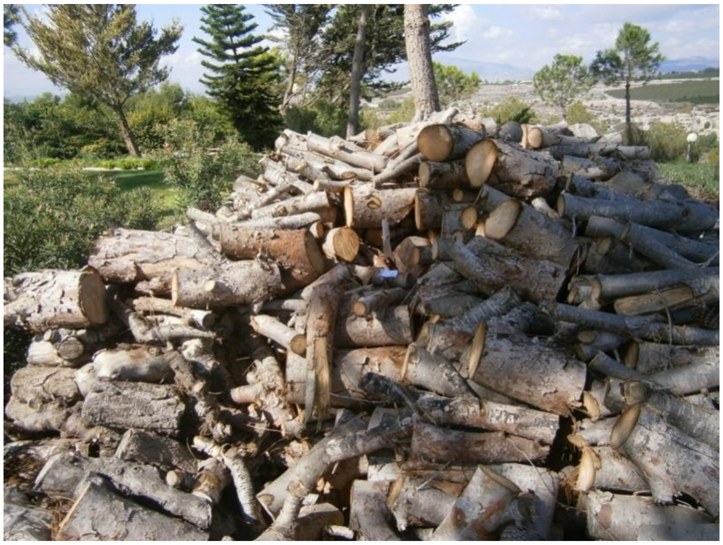

Photo 04: Old wood pile (site 6).

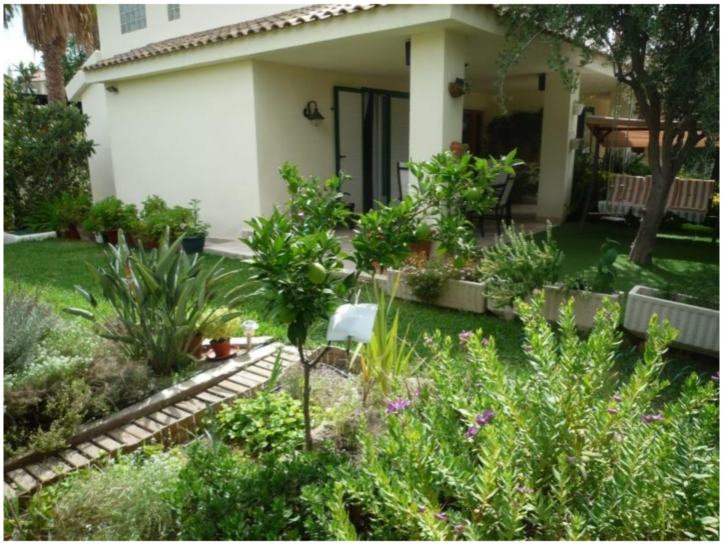

Photo 05: Garden (site 15).

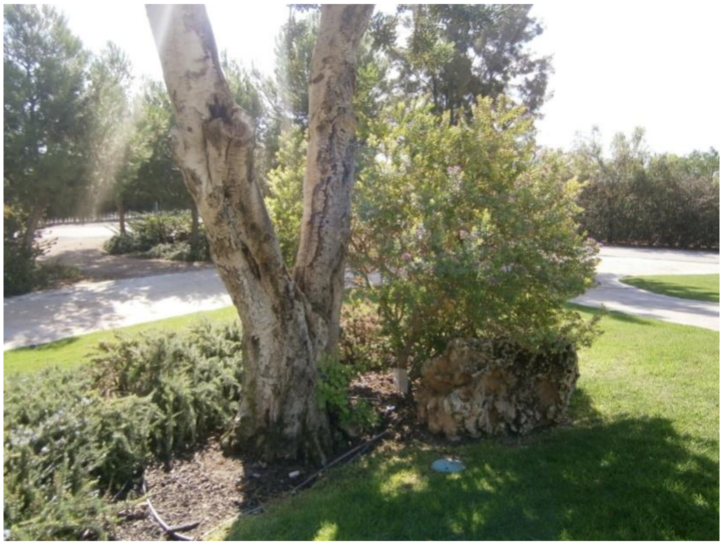

Photo 06: Garden (site 7).

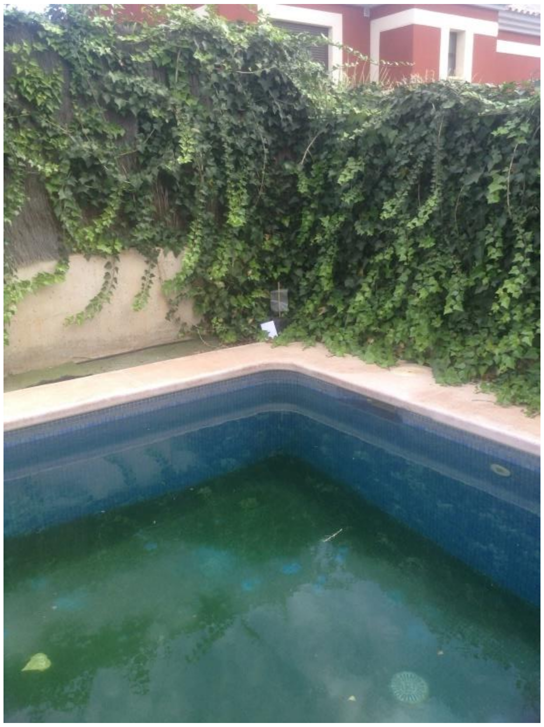

Photo 07: Garden (site 7).

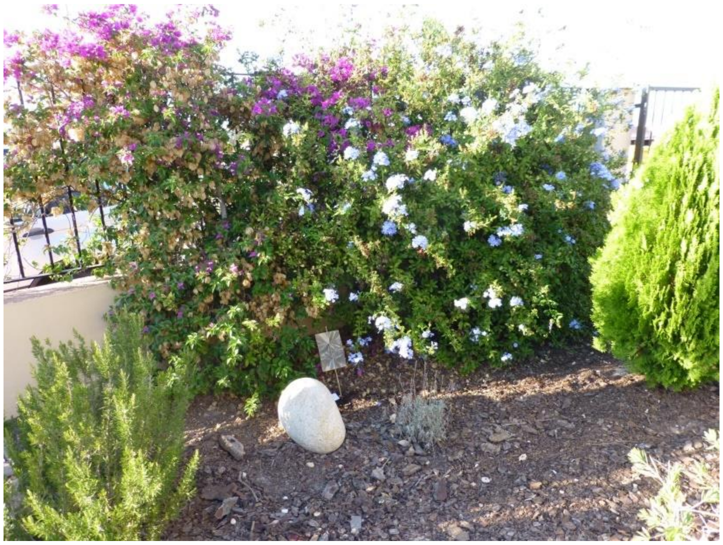

Photo 08: Garden (site 3).

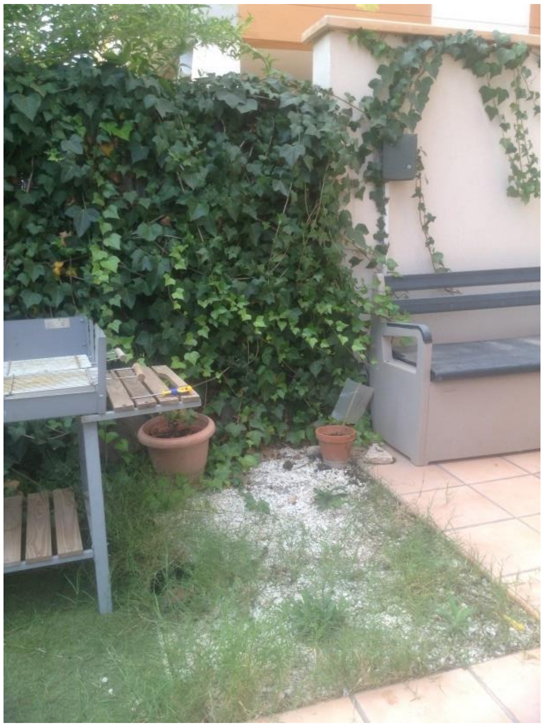

Photo 09: Garden (site 16).

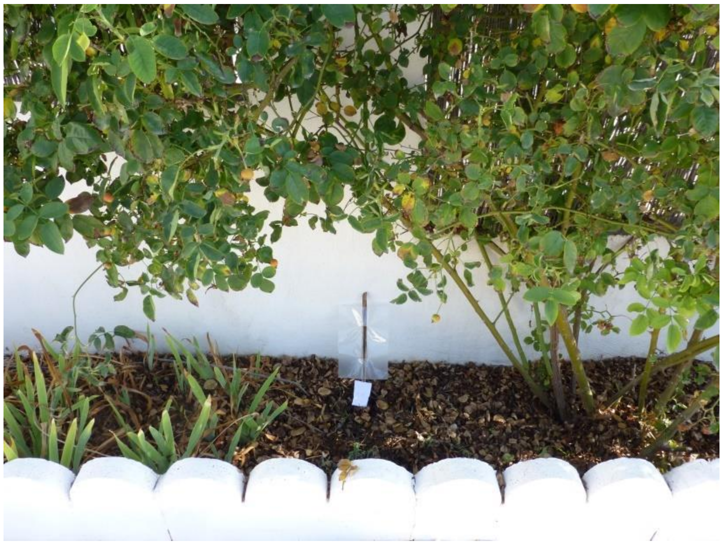

Photo 10: Garden (site 2).

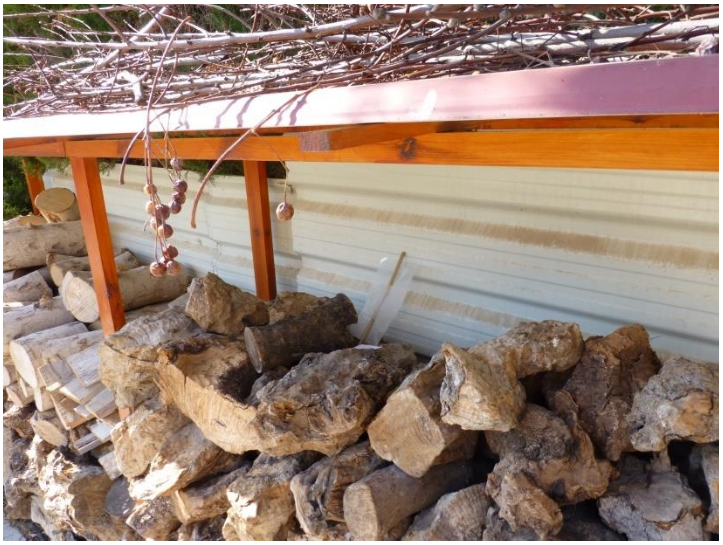

Photo 11: Wood pile (site 2).

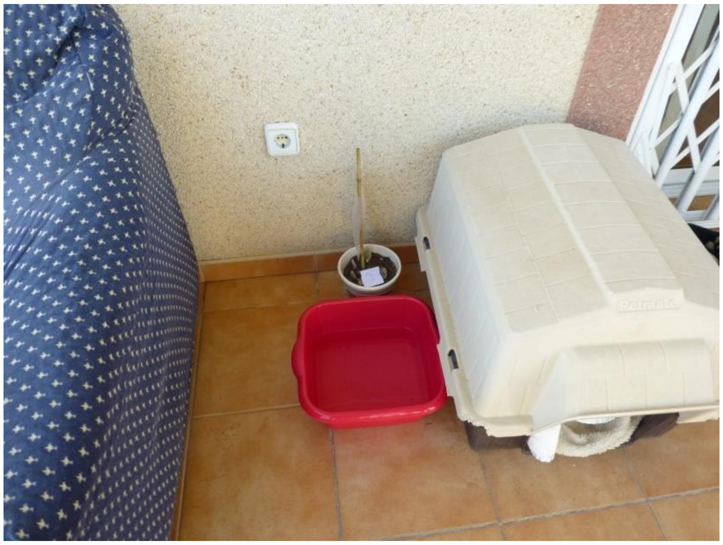

Photo 12: Porsche (site 2).

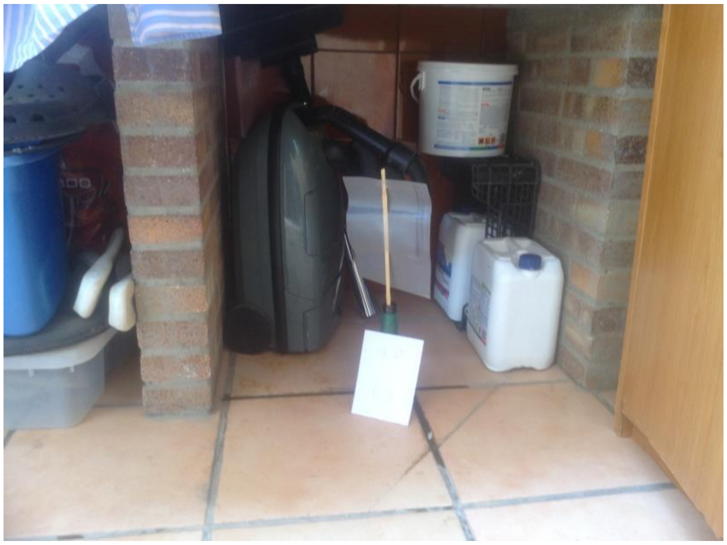

Photo 13: Open accessory room (site 8).

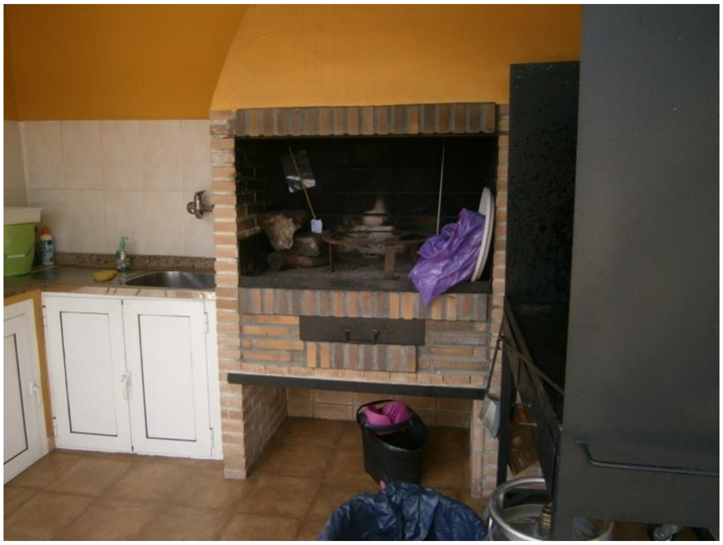

Photo 14: Open barbeque room (site 5).

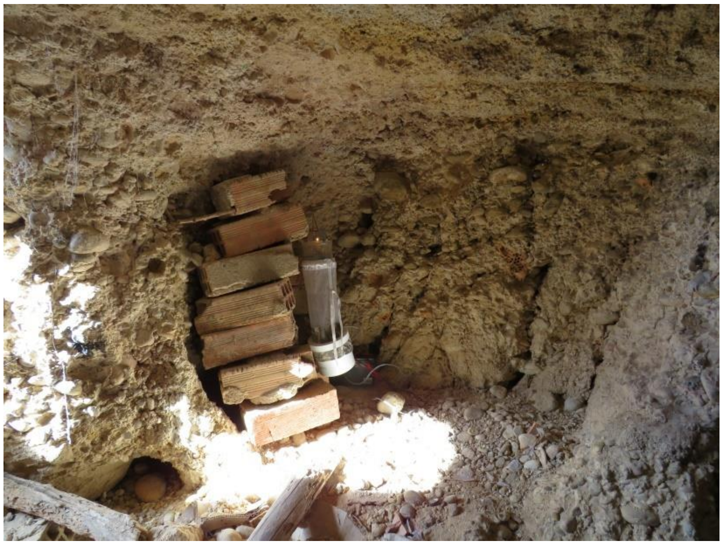

Photo 16: Open cave (site 1).

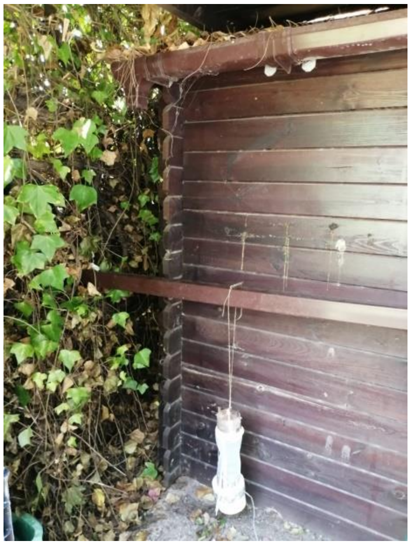

Photo 17: Site 3.

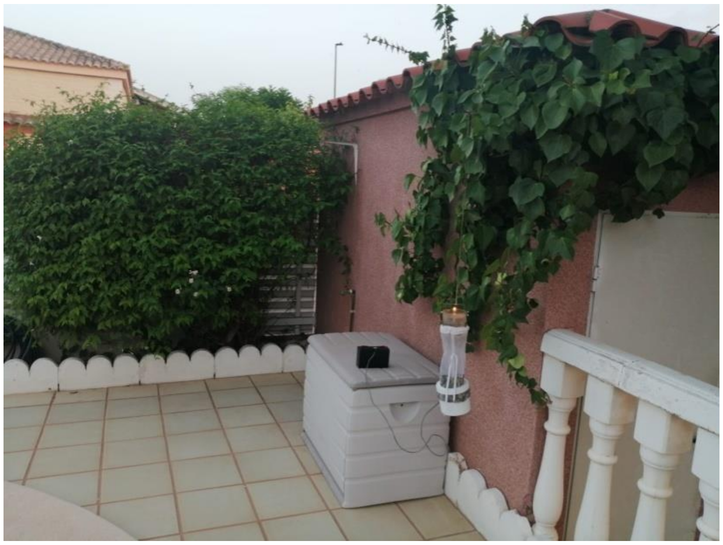

Photo 18: Site 2.
